# Exploring perceptions in the management and treatment of polycythaemia vera in the UK

**DOI:** 10.1007/s00277-025-06352-8

**Published:** 2025-04-09

**Authors:** Becky Bagnall, Claire Woodley, Rozinder Bains, Katherine Gibson, Amir Nawaz, Jacqueline Ryan, Mary Frances McMullin

**Affiliations:** 1 North Bristol NHS Trust, Bristol, UK; 2 Guy’s and St Thomas’ NHS Foundation Trust, London, UK; 3 Novartis Pharmaceuticals UK Ltd, London, UK; 4 Queen’s University Belfast, Belfast, Northern Ireland

**Keywords:** Diagnosis, Management, Polycythaemia vera, Recommendations

## Abstract

Approximately 1140 people are diagnosed with polycythaemia vera (PV) annually in the United Kingdom (UK). Adherence to the British Society of Haematology (BSH) guidelines for PV diagnosis and management is not well understood. To explore UK’s PV diagnosis, management practices and unmet needs. A structured survey, co-developed with a UK haematology consultant, an advanced nurse practitioner and a pharmacist, was completed by 57/332 invited healthcare practitioners from July to October 2023 through 1:1 interviews conducted by Novartis Medical Science Liaisons. Results were analysed descriptively. Most respondents (68%) follow the BSH 2018 guidelines for diagnosing PV. Treatment goals are to reduce thromboembolic event risk and control haematocrit and symptoms. Most patients (68%) were receiving cytoreductive therapy (typically first-line hydroxycarbamide); 28% received antiplatelet medication and/or venesection alone. Stable patients are usually monitored every 3 months through telephone (68%), increasing to monthly when uncontrolled, mainly in-person (54%). General practitioners (56%) manage cardiovascular risks, but there is doubt over referral response. All respondents monitor symptoms, with only 19% regularly using MPN10. The greatest educational need was identifying hydroxycarbamide resistance and intolerance (58%). This survey offers insights into therapeutic approaches and areas for improvement in the UK’s PV clinical practice.

## Introduction

Polycythaemia vera (PV) is a Janus kinase 2 (*JAK2*)–mutated myeloproliferative neoplasm (MPN) characterised by clonal erythrocytosis, with increased risk of thrombosis and progression to myelofibrosis or acute myeloid leukaemia [1]. PV has an annual estimated incidence of 1140 newly diagnosed patients in the United Kingdom (UK) [2]. PV typically presents at a median age of 61 years [3], with a slight male predominance [4]. Median survival is around 14.1 years but exceeds 35 years for patients aged ≤ 40 years [1, 3]. While patients with PV may present with thromboembolic events or disease-related symptoms that significantly impact quality of life (QoL), some may be asymptomatic and identified incidentally through a full blood count (FBC) [4]. Nearly all patients (95%) with PV have activating *JAK2* driver mutations (V617F and exon 12 mutations) in haematopoietic stem cells [5].

According to the International Consensus Classification, the World Health Organization and British Society for Haematology guidelines, the major diagnostic criteria include elevated haemoglobin concentration and/or elevated haematocrit value, presence of JAK2 V617F or JAK2 exon 12 mutation and trilineage growth (panmyelosis) with pleomorphic mature megakaryocytes [4, 6–8].

Current treatment goals are to reduce the risk of thromboembolic events [4], which are the major cause of morbidity and mortality in PV [9], as well as alleviate symptoms, improve survival and minimise risk of transformation to myelofibrosis and acute leukaemia [4]. Periodic venesection/phlebotomy, with a HCT target of < 45%, combined with low-dose aspirin in the absence of contraindications, are the initial therapeutic options for low-risk patients [1]. Cytoreductive therapy is typically reserved for patients with high-risk (aged > 65 years and/or a history of thrombosis) disease, with hydroxycarbamide (HC) and pegylated interferon-α as first-line drugs, ruxolitinib as a second-line option and busulfan as a third-line option [4].

First published in 2005 [10] and updated in 2019 [4], the British Society for Haematology’s guidelines for diagnosing and managing PV are widely accepted across the UK, although the extent of their adherence is not fully understood. ‘Exploring Perceptions In Management And Treatment Of Polycythaemia Vera in the UK’ (PV-PINPOINT), a nationwide survey of various healthcare practitioners (HCPs), sought to understand current practices and unmet clinical and HCP educational needs in PV in the UK. This report presents insights into current practices regarding PV diagnosis, treatment and management and outlines areas requiring further education and better usage of existing guidance to optimise patient outcomes.

## Methods

### Survey design and dissemination

A structured survey was co-developed with an expert steering committee of a UK haematology consultant, an advanced nurse practitioner and a pharmacist, ensuring the survey was clinically accurate and relevant to the healthcare community. The online survey included 57 mandatory multiple-choice questions, some with free-text fields, partitioned into five sections: introduction, diagnosis, treatment, monitoring and education and support (Supplementary Material). To qualify for the study, HCPs must have managed > 5 patients with PV in the previous 12 months and be directly involved in the overall treatment and management of patients with PV.

Novartis Medical Science Liaisons (MSLs) invited 332 HCPs from district general hospitals and tertiary care centres to participate in the study to ensure a diverse representation of background characteristics. The survey was conducted in accordance with the British Healthcare Business Intelligence Association guidance for the conduct of market research.

### Data analysis

The final dataset used for analysis contained responses to all questions (no missing responses). The results were descriptively analysed by Adelphi Research (Cheshire, UK). Categorical variables were presented as frequency and percentages (with a denominator of 57, unless otherwise stated).

For free-text responses, individual responses were analysed to identify key themes and categorised by coding. Coding was generated by a third-party data service provider and verified by ADELPHI Research.

Subgroups were investigated by speciality, centre (large teaching versus local), region and caseload.

## Results

Between July and October 2023, 57 of 332 HCPs, including consultants (*n* = 37), clinical nurse specialists (*n* = 10), advanced nurse practitioners (*n* = 5) and prescribing pharmacists (*n* = 5), completed the 45-minute digital survey through 1:1 interviews with MSLs. The respondents offered a geographically diverse representation of the UK coming from secondary care (local/district general hospital [DGH]; *n* = 22) and tertiary care (*n* = 35) centres across England (*n* = 47), Scotland (*n* = 5), Wales (*n* = 2) and Northern Ireland (*n* = 3). The analysis of the survey responses was divided into four distinct sections, reflecting the survey structure.

### Guidelines and diagnosis

Most HCPs (39/57, 68%) reported daily practice in line with the BSH 2018 guidelines for diagnosis, risk stratification and treatment [4], and some followed the WHO 2016 guidelines (8/57, 14%) [8]. For PV diagnosis, most HCPs reported using serum erythropoietin (EPO) level (44/57, 77%), alongside an FBC (49/57, 86%) and peripheral blood *JAK2 V617F* mutational analysis (49/57, 86%). Bone marrow biopsy was used by 37% (21/57) of. HCPs with a high caseload (> 150 patients, *n* = 9) all consistently perform a FBC and *JAK2 V617F* mutation analysis. At diagnosis, more than half (20/37, 54%) of the consultants also request a next-generation sequencing (NGS) panel test for a proportion of their patients (typically younger patients [35%] or those at greater risk of progression [30%], followed by patients with atypical features/without classic mutations [20%], patients without a clear diagnosis [15%] and patients negative for *JAK2/JAKV617F* [15%]). Those working in academic centres were more likely to use NGS (18/26, 69%).

Due to limited capacity, HCPs reported that the multidisciplinary teams (MDTs) are likely to prioritise patients with complex (40/57, 70%) and high-risk (38/57, 67%) conditions, and not all patient categories are routinely discussed. At least half of the HCPs discuss patients at MDT meetings regularly throughout their treatment journey, particularly in the case of transformation (55/57, 96%). Other timepoints include, but are not limited to, when the patients experience resistance in first-line therapy (35/57, 61%) and second-line therapy (37/57, 65%) or those with complex disease (common definitions including those with pruritus/itching and thrombotic events; 36/57, 63%). When comparing by clinical centre type (academic/specialist versus local/DGH), HCPs as part of an MDT discuss patients who are resistant/intolerant to first-line (51% [18/35] versus 77% [17/22], and 49% [17/35] versus 73% [16/22], respectively) and second-line (57% [20/35] versus 77% [17/22], and 51% [18/35] versus 68% [15/22], respectively) therapies, along with patients with complex conditions (57% [20/35] versus 73% [16/22], respectively) more regularly in a DGH setting.

### Treatment

**Fig. 1:**
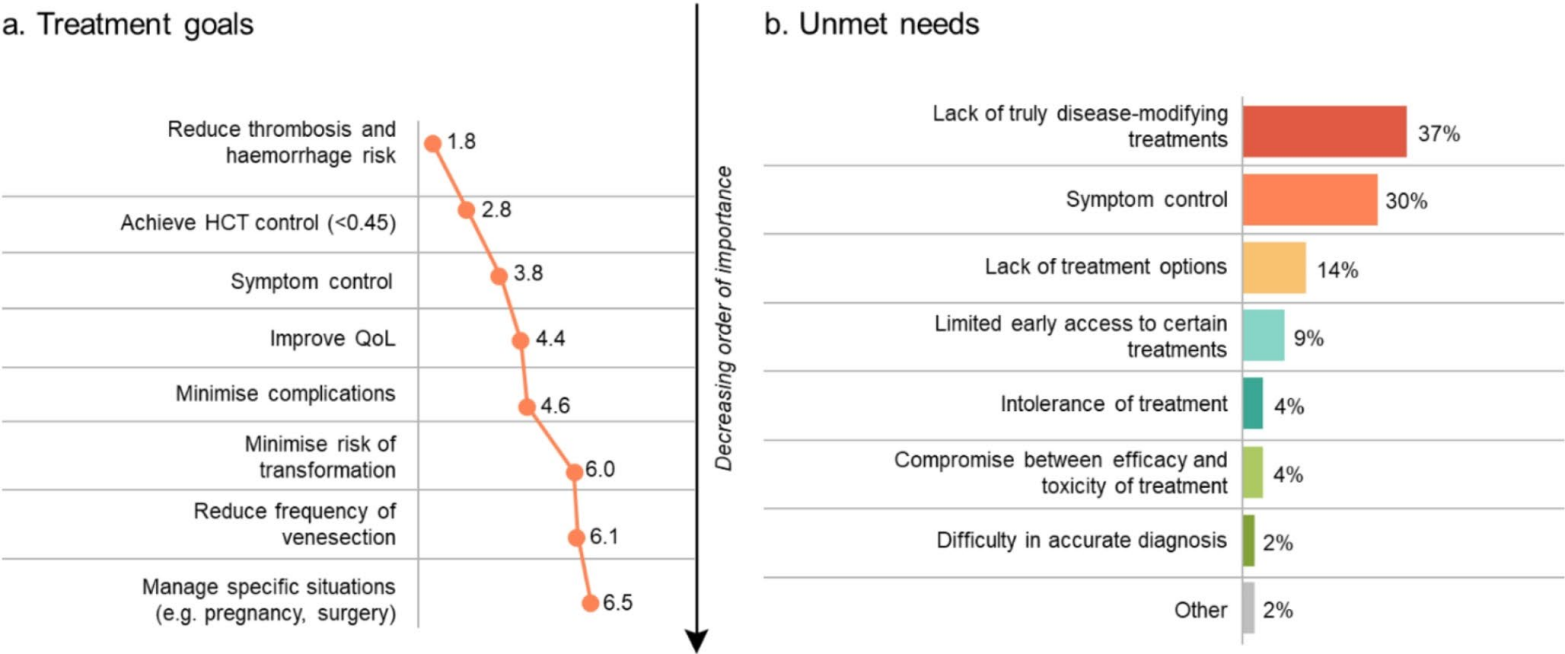
Treatment goals and unmet needs of patients with PV. **a**) Mean ranking 1–8 (1 = most important, 8 = least important). **b**) % of HCPs ranking as #1. HCP, healthcare professional; HCT, haematocrit; QoL, quality of life

Using a mean ranking of 1–8 (1 = most important; 8 = least important), the survey revealed that the key treatment goals included reduction of thrombosis and haemorrhage risk (1.8), HCT control to < 0.45 (2.8) and symptom control (3.8; Fig. 1a). The greatest unmet needs of patients with PV were lack of truly disease-modifying treatments (21/57, 37%) and symptom control (17/57, 30%). Less commonly prioritised unmet needs included lack of treatment options, limited early access to certain treatments, intolerance of treatment, compromise between efficacy and toxicity of treatment and difficulty in accurate diagnosis (Fig. 1b).

For patients receiving first-line treatment, sub-optimal control was commonly defined by increased HCT levels of > 0.45 (53/57, 93%) and a worsening of symptoms (44/57, 77%); 32% (18/57) of HCPs reported that 21-40% of their patients were switched to an alternative treatment for this reason. Most HCPs (51/57, 90%) reported intervention for almost all or all patients with HCT > 0.45. The reasons for not intervening included borderline HCT (equal to 0.45; 15/57, 26%); frail/elderly patients (10/57, 18%); patient preference (6/57, 11%), anaemia (6/57, 11%), intolerant patients (6/57, 11%), unwell/infected patients (3/57, 5%) and dehydration (3/57, 5%).

HCPs reported that most patients with PV (68%) were receiving cytoreductive therapy– with 52%, 12% and 4% receiving first-line, second-line and third-line therapies, respectively. Treatment with antiplatelet agents and/or venesection alone was less common (28%) and was usually due to low-risk status (52/57, 91%) or patient refusal (43/57, 75%). HCPs reported only 1% of patients were reported as not receiving treatment.

Most HCPs (44/57, 77%) chose HC as first-line cytoreductive therapy, followed by interferon (13/57, 22%) for their patients with PV. HC was mainly prescribed to patients with a prior history of severe depression (36/57, 63%), whereas interferon was often used for patients with a history of skin cancer (32/53, 60%) or planning a family (46/53, 87%). HCPs in a DGH setting were more likely to prescribe HC versus specialist centres (86% [19/22] versus 72% [25/35]). Signs of HC resistance/intolerance were reviewed at every clinic visit by most HCPs (46/57, 81%). Non-consultants (19/20, 95%) were more likely to review resistance/intolerance at every clinic meeting (27/37, 73%). The most common indicators of resistance/intolerance to HC were presence of ulcers (98%), neutropenia (93%) and venesection need (82%). Some HCPs reported reviewing HC resistance/intolerance every 3 months (9/57, 16%). For patients intolerant or resistant to HC, most HCPs favoured interferon (49/57, 86%), followed by JAK inhibitors (35/57, 61%), and busulfan (21/57, 37%). JAK inhibitors were used more in academic/specialist centres (25/35, 71%) versus local/DGH settings (10/22, 45%). For HCPs with the highest caseload (> 200), various treatments were used, including interferon (9/10, 90%), JAK inhibitors (7/10, 70%) and busulfan (6/10, 60%).

**Fig. 2:**
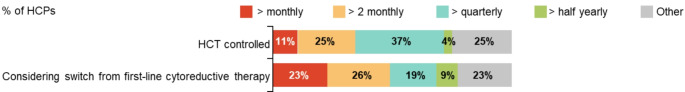
Venesection frequency threshold. HCPs were asked the following question: What is your venesection frequency threshold for considering switching to an alternative treatment option for patients on a first-line cytoreductive therapy? HCP, healthcare professional; HCT, haematocrit

HCPs’ frequency threshold for venesection was variable. In patients with HCT-controlled disease, 11% (6/57) of HCPs reported their thresholds to be more than once a month and 25% (14/57) reported more than every 2 months. When a switch from first-line cytoreductive treatment was being considered, 49% (28/57) of HCPs reported a venesection frequency threshold of more than every 2 months, while 23% (13/57) reported more than once a month (Fig. 2).

### Monitoring and management

Independent of patients’ cytoreductive therapy status, most HCPs generally monitored stable patients every 3 months and uncontrolled patients monthly. Among patients receiving cytoreductive therapy, 70% (40/57) of HCPs monitored stable patients on a 3-monthly basis and 67% (38/57) of HCPs monitored uncontrolled patients monthly (Table 1a). Nurses were identified as having a leading role in managing stable patients (31/57, 54%) and often responsible for leading the PV clinics (37/57, 65%; Table 1b). For stable patients on cytoreductive therapy, 68% of HCPs (39/57) managed these patients by telephone, while 54% (31 of 57) of HCPS managed uncontrolled patients in-person (Table 1c). Where separate non-consultant-led (nurse- or pharmacist-led clinics, independent of consultants) and consultant-led clinics were in place, 52% (22/42) reported using guidelines/protocols for referral that were most common local guidelines (41%) or the 2018 BSH guidelines (32%). FBCs are universally performed. Only 9% (5/57) of HCPs use sequential testing of *JAKV617F* allele burden, mainly only in patients receiving interferon.

**Table 1:** Monitoring and management of stable and uncontrolled patients

**a. Patient monitoring frequency**			
		On cytoreductive therapy	Not on cytoreductive therapy
Stable patients, n (%)	Monthly	0 (0)	0 (0)
	3 monthly	40 (70)	26 (46)
	4 monthly	12 (21)	9 (16)
	6 monthly	2 (4)	14 (25)
	Ad hoc	0 (0)	1 (2)
	Other	3 (5)	7 (12)
Uncontrolled patients, n (%)	Monthly	38 (67)	31 (54)
	3 monthly	1 (2)	5 (9)
	4 monthly	0 (0)	0 (0)
	6 monthly	0 (0)	0 (0)
	Ad hoc	3 (5)	4 (7)
	Other	15 (26)	17 (30)
**b. Specialities responsible for stable patient management and leading PV clinics**			
		**PV clinic lead**	**Management of stable patients**
n (%)	Nurse	37 (65)	31 (54)
	Pharmacist	19 (33)	3 (5)
	Physician associate	2 (4)	1 (2)
	Other senior doctor	12 (21)	0 (0)
	Registrar	12 (21)	0 (0)
	Senior house officer	2 (4)	0 (0)
	Other	10 (18)	22 (39)
**c. Consultation channels**			
		**On cytoreductive therapy**	**Not on cytoreductive therapy**
Stable patients, n (%)	In-person	1 (2)	4 (7)
	Telephone	39 (68)	35 (61)
	Other– in person and phone	10 (18)	10 (18)
	Other	7 (12)	8 (14)
Uncontrolled patients, n (%)	In-person	31 (54)	29 (51)
	Telephone	11 (19)	12 (21)
	Other– in person and phone	13 (23)	13 (23)
	Other	2 (4)	3 (5)

n, number of participants; PV, polycythaemia vera

#### Cardiovascular risk monitoring

**Fig. 3:**
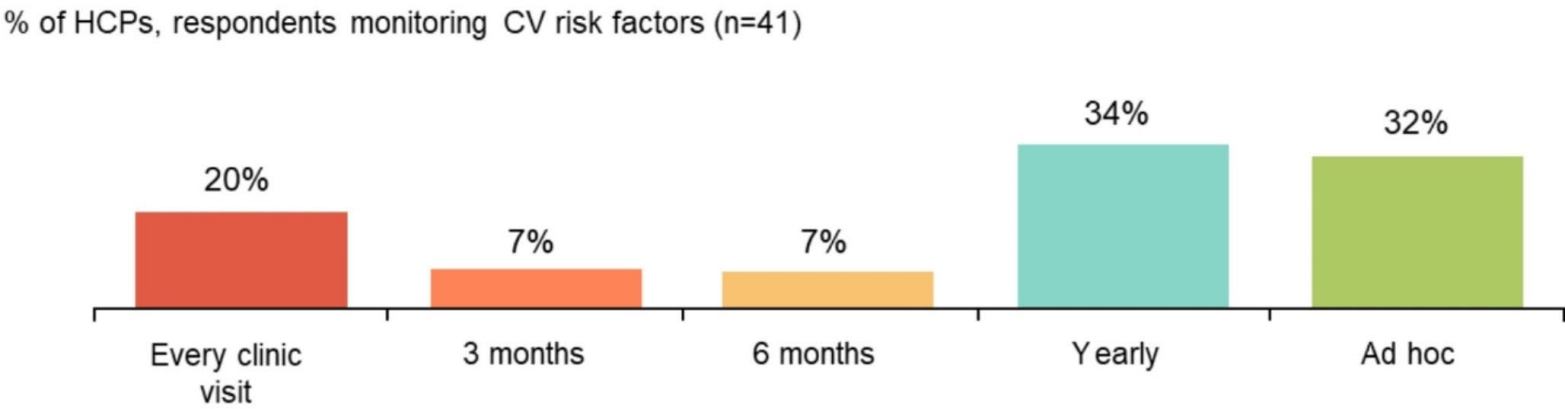
Frequency of CV risk factor monitoring in clinic. CV, cardiovascular; HCP, healthcare professional

Primary care general practitioners (GPs; 56%) were the main HCPs responsible for ongoing evaluation and management of cardiovascular (CV) disease. However, only 16% (9/57) of HCPs were ‘extremely confident’ that a GP will act on a CV assessment referral. In clinic, CV risk factors were monitored at every visit by 20% (8/41) of HCPs, annually by 34% (14/41) of HCPs and on an ad hoc basis by 32% (13/41; Fig. 3). While smoking and hypertension are the most commonly monitored CV risk factors (54% [31/57] and 47% [27/57] in clinics, respectively), 28% (16/57) of HCPs did not monitor these factors and instead consider this to be the responsibility of GPs.

#### Symptom monitoring

**Fig. 4:**
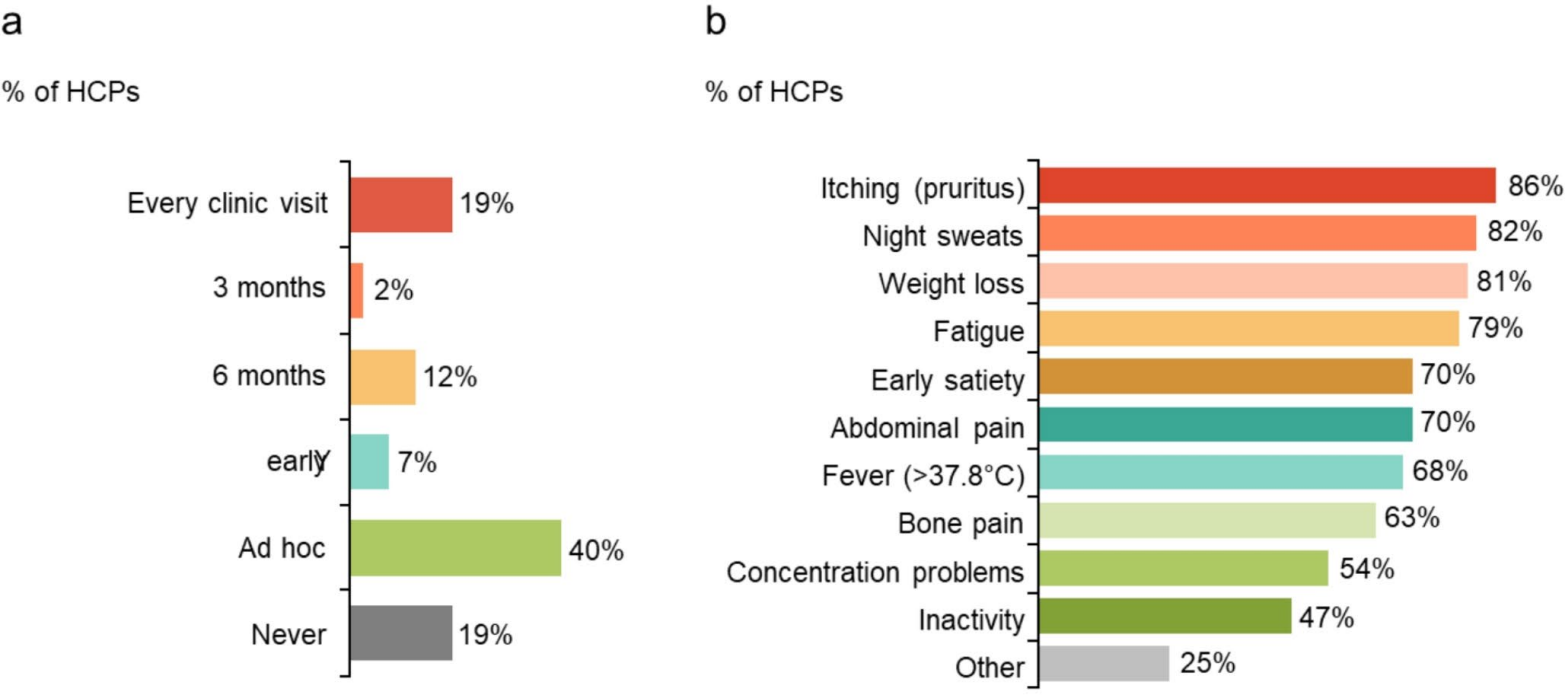
(**a**) Frequency of clinical MPN10 use and (**b**) symptoms monitored. HCP, healthcare professional; MPN10, Myeloproliferative Neoplasm Symptom Assessment

Despite symptom control being identified as the third most important treatment goal and all HCPs actively monitoring patients with PV for symptoms, only 19% (11/57) used the MPN10 (assessment tool for symptoms) at every clinic visit, although 81% (46/57) cited the use of MPN10 (Fig. 4a) and the specific symptoms monitored varied (Fig. 4b). HCPs with the highest caseload (> 200) are more likely to track symptoms to initiate treatment (8/10, 80%) than those with the lowest patient caseload (< 50; 2/7, 29%). Furthermore, HCPs in academic/specialist centres are more likely to track some symptoms versus those in DGH settings, including bone pain (74% [26/35] versus 45% [10/22]) and concentration problems (63% [22/35] versus 41% [9/22]).

#### Education and support

**Fig. 5:**
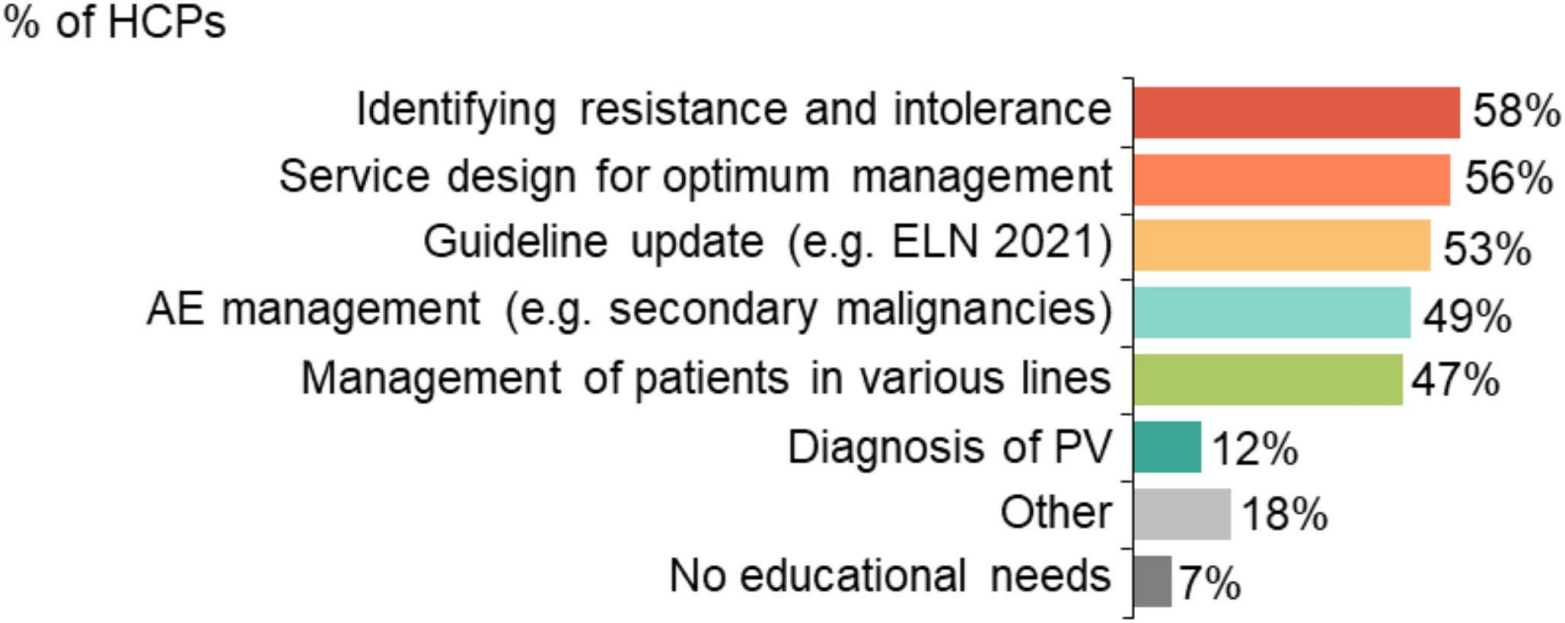
Further education requirements. ELN, European LeukemiaNet; PV, polycythaemia vera

Most HCPs (48/53, 91%) reported that clinical nurse specialists would benefit from additional training and education, followed by junior doctors (35/53, 66%), pharmacists (33/53, 62%) and consultants (31/53, 58%). Identification of resistance and intolerance was cited as the greatest educational need by 58% (33/57) of HCPs, followed by service design for optimum management (32/57, 56%) and guideline updates (30/57, 53%; Fig. 5).

## Discussion

The key findings from this study suggest that, in the UK, PV diagnosis and management are largely aligned with the current BSH guidelines for PV [4]. This nationwide study aimed to understand the present management and landscape for PV in the UK. Despite two-thirds of HCPs adhering to the BSH 2018 guidelines [4], our survey identifies several key findings and areas for improvement.

### Diagnosis

Most HCPs followed a specific trend in diagnosing PV, which included the use of serum EPO, FBC and peripheral blood for *JAK2 V617F* mutational analysis. Bone marrow biopsy was used less frequently in diagnosis in this survey compared with an Italian survey on clinical practice in MPNs (37% versus 47.5%) [11]. HCPs managing larger caseloads tended to rely on FBCs and *JAK2 V617F* mutational analysis. Interestingly, over half of them incorporated NGS into their diagnostic process, especially those working in academic centres, despite it not being a requirement for PV diagnosis. Not all HCPs discuss all newly diagnosed patients with PV at MDT meetings, despite recommendations from the BSH 2018 guidelines [4], indicating the need for greater guidance awareness. Patients were often discussed at various disease stages, with more focus on complex cases, treatment resistance and instances of transformation. HCPs in smaller centres and DGHs had more frequent discussions about patients who exhibited treatment resistance and intolerance. No notable differences were observed for practices between consultants and nurses.

### Treatment

All HCPs aim to decrease the risk of thrombosis and haemorrhage, indicating shared treatment goals. The reported lack of truly disease-modifying treatments as the greatest unmet need underscores the necessity for further research and treatment optimisation and is beyond the scope of the present publication. Consistent with the BSH guidelines [4], most HCPs intervene for almost all patients with HCT > 0.45. More than two-thirds of patients were receiving cytoreductive therapy, with HC being the most common treatment. In the Italian survey on clinical practice in MPNs, nearly all HCPs (96.7%) preferred HC as the first-line cytoreductive therapy of choice for patients for PV, which was higher than that observed in this study (77%) [11]. However, in this survey, interferon became the primary treatment option for patients who developed resistance or intolerance to HC. Ruxolitinib was the preferred second-line therapy of choice in the Italian survey, however, the reason for treatment switch was not disclosed [11]. Low-risk status and patient refusal were the key reasons for the use of only anti-platelet agent/venesection only. HCPs also reported variability in venesection frequency threshold, irrespective of treatment and disease control. This variability likely exists due to differences in patient disease journeys (early versus prolonged disease) and may also be influenced by the frequency of HCP visit. Existing guidance from the European LeukaemiaNet 2021 recommends switching treatment for patients receiving HC if they require more than 6 venesections per year [12]. There is a need for broader education to clarify when treatment switches are required, considering venesection is time consuming, resource-intensive [13], and its higher frequency may lower patients’ QoL, as highlighted in the MPN Landmark Survey [14]. The same guidance also recommends switching treatment for patients receiving HC when there is an insufficient clinical response (at ≥ 1.5 g per day for at least ≥ 4 months and without reporting intolerance) or due to intolerance to HC (due to grade 3–4 or prolonged grade 2 non-haematological toxicity, haematological toxicity [haemoglobin < 100 g/L, platelet count < 100 × 10^9^ cells per L, or neutrophil count < 1 × 10^9^ cells per L], or development of non-melanoma skin cancer or vascular events) [12]. Further education across HCPs managing PV is needed, particularly in recognising HC resistance and intolerance, considering not all HCPs monitor this at every visit.

### Monitoring and management

Nurses are often the primary clinician managing stable patients and frequently lead PV clinics. Stable patients are generally monitored every 3 months, mostly through telephone clinics, regardless of treatment. At diagnosis, a patient’s CV risk is assessed by consultants, after which they are referred to GPs for continuous monitoring. However, the frequency of monitoring in the clinic varies, highlighting a potential need for standardisation. This standardisation is likely to improve confidence in how GPs respond to referrals. Monitoring of CV risk was variable, with nearly a third of respondents monitoring on an ad hoc basis. Furthermore, less than one-third of patients were monitored for smoking and hypertension (the most common CV risk factors) in the clinic. Control of CV risk factors alongside treatment may improve patient outcomes, although further research is required to confirm this [15]. A recent study utilising a novel natural language processing model for CV risk assessment enabled the processing and analysis of 24,155 documents from patients with PV and essential thrombocythaemia to inform patient management and risk prediction for CV events [16]. Although most GPs supported the ongoing management of CV risk, HCPs had limited confidence in these referrals, highlighting a need to improve collaboration between HCPs and GPs for CV risk assessment and management. Existing CV monitoring for GPs includes health checks provided by the National Health Service for people aged 40–74 years, which can indicate whether a patient is at risk of heart disease and ongoing management [17]. In October 2021, the development of the Network Contract Directed Enhanced Service provided requirements for the delivery of a CV disease prevention and diagnosis service by primary care networks, with the aim to reduce the impact of atrial fibrillation, hypertension and cholesterol [18]. However, some differences in CV monitoring may exist across nations.

There is also scope to further standardise the monitoring of key PV symptoms, considering that changes in symptoms could indicate disease progression, which persist despite control of blood counts [19] and prior treatment (HC) or in those with clinical features, such as splenomegaly [20] and can impact QoL [13, 21]. Despite symptom control being ranked as the third most important treatment goal in PV and the second greatest unmet need, the approach to symptom monitoring was inconsistent. This could be improved by utilising a validated symptom assessment tool, such as MPN10, more extensively, and further understanding of factors that influence or prevent regular use in clinical visits is necessary. Patients may also benefit from the development of a specific symptom questionnaire for PV, which could include monitoring of possible microvascular symptoms, such as erythromelalgia, headache, visual and mood disturbances, paraesthesia and sexual dysfunctions [22], as well as monitoring of constitutional symptoms. Understanding of the potential value of this questionnaire to patients and its development requires further study.

### Educational needs

All HCPs would benefit from additional training and education, in particular clinical nurse specialists, registrars/junior doctors and pharmacists as MPN clinics evolve. Interestingly, despite intolerance of treatment being ranked as a lower priority unmet need among respondents, identification of HC resistance and intolerance was cited as the greatest educational need for managing PV. This would help HCPs to understand as early as possible when a treatment change is necessary. Further understanding of service design and guideline updates will likely contribute to an optimised and standardised management of PV.

### Limitations

The response rate for the survey was relatively low, however comparable to the response rate on another national MPN survey [11]. This low response rate may be attributed to several factors including the high workloads and time constraints for HCPs. The specialty, primary care setting and geographical distribution of responders to this survey ensures that the results reported provide an important view of clinical practice within the UK. A limitation of the survey includes analyses based on self-reported practice versus observed and may be subject to recall bias. Potential bias introduced by the Novartis medical team conducting the interviews should also be considered.

### Expert recommendations

- All HCPs can benefit from training, in particular, clinical nurse specialists, pharmacists and junior doctors.
- MDT discussion for all patients at diagnosis and for treatment switching (as minimum).
- Provide clear definitions of HC resistance and intolerance in patients.
- Establish a more structured service design, for example, an MPN clinic with links between doctors and nurses.
- Routine and regular reference and utilisation of the BSH guidelines for guidance on optimal management of patients with PV.
- Routine and regular symptom monitoring, utilising MPN10.
- Routine and regular CV monitoring for all patients with PV.

## Conclusion

This survey offers critical insights into the current therapeutic approaches and identifies potential areas for advancement within the UK’s current clinical practice and treatment landscape of PV.

## Data Availability

Data collection was undertaken by Adelphi Research as part of a survey conducted in collaboration with Novartis Pharmaceuticals UK Ltd, entitled PV-PINPOINT. All data supporting the findings of this study are the intellectual property of Novartis Pharmaceuticals UK. All requests for access should be addressed directly to Rozinder Bains < rozinder.bains@novartis.com>.
